# Mixed Connective Tissue Disease as Different Entity: Global Methylation Aspect

**DOI:** 10.3390/ijms242015495

**Published:** 2023-10-23

**Authors:** Gabriela Filipowicz, Anna Wajda, Barbara Stypińska, Tomasz Kmiołek, Anna Felis-Giemza, Sandra Stańczyk, Zenobia Czuszyńska, Marcela Walczyk, Marzena Olesińska, Agnieszka Paradowska-Gorycka

**Affiliations:** 1 Department of Molecular Biology, National Institute of Geriatrics, Rheumatology and Rehabilitation, Spartanska 1, 02-637 Warsaw, Poland; gzajac@vp.pl (G.F.);; 2 Biologic Therapy Center, National Institute of Geriatrics, Rheumatology and Rehabilitation, Spartanska 1, 02-637 Warsaw, Polandsandra.stanczyk@spartanska.pl (S.S.); 3 Department of Rheumatology, Clinical Immunology, Geriatrics and Internal Medicine, Medical University of Gdansk, Smulochowskiego 17, 80-214 Gdansk, Poland; 4 Department of Connective Tissue Diseases, National Institute of Geriatrics, Rheumatology and Rehabilitation, Spartanska 1, 02-637 Warsaw, Poland

**Keywords:** ACTDs, MCTD, SSc, SLE, epigenetics, DNA methylation

## Abstract

Mixed connective tissue disease (MCTD) is a very rare disorder that belongs in the rare and clinically multifactorial groups of diseases. The pathogenesis of MCTD is still unclear. The best understood epigenetic alteration is DNA methylation whose role is to regulate gene expression. In the literature, there are ever-increasing assumptions that DNA methylation can be one of the possible reasons for the development of Autoimmune Connective Tissue Diseases (ACTDs) such as systemic sclerosis (SSc) and systemic lupus erythematosus (SLE). The aim of this study was to define the global DNA methylation changes between MCTD and other ACTDs patients in whole blood samples. The study included 54 MCTD patients, 43 SSc patients, 45 SLE patients, and 43 healthy donors (HC). The global DNA methylation level was measured by ELISA. Although the global DNA methylation was not significantly different between MCTD and control, we observed that hypomethylation distinguishes the MCTD patients from the SSc and SLE patients. The present analysis revealed a statistically significant difference of global methylation between SLE and MCTD (*p* < 0.001), SLE and HC (*p* = 0.008), SSc and MCTD (*p ≤* 0.001), and SSc and HC (*p* < 0.001), but neither between MCTD and HC (*p* = 0.09) nor SSc and SLE (*p* = 0.08). The highest % of global methylation (median, IQR) has been observed in the group of patients with SLE [0.73 (0.43, 1.22] and SSc [0,91 (0.59, 1.50)], whereas in the MCTD [0.29 (0.20, 0.54)], patients and healthy subjects [0.51 (0.24, 0.70)] were comparable. In addition, our study provided evidence of different levels of global DNA methylation between the SSc subtypes (*p* = 0.01). Our study showed that patients with limited SSc had a significantly higher global methylation level when compared to diffuse SSc. Our data has shown that the level of global DNA methylation may not be a good diagnostic marker to distinguish MCTD from other ACTDs. Our research provides the groundwork for a more detailed examination of the significance of global DNA methylation as a distinguishing factor in patients with MCTD compared to other ACTDs patients.

## 1. Introduction

Autoimmune Connective Tissue Diseases (ACTDs) are rare and clinically multifactorial groups of diseases, varying in terms of the aspect of specific autoreactive immune cells and autoantibodies produced, organs or tissues attacked, and the clinical phenotype. The most frequent diseases of ACTDs are systemic lupus erythematosus (SLE), rheumatoid arthritis (RA), relatively rare systemic sclerosis (SSc), and mixed connective tissue disease (MCTD). The pathogenesis of ACTDs is so far unclear. In environmental factors such as diet and drugs, genetics and epigenetics are recognized. However, some common ACTDs features related to etiology are recognized, which may consequently result in similar treatment approaches [1,2].

MCTD is a very rare disorder with the so-called overlap syndromes, which means MCTD combines the similar clinical symptoms of SSc, SLE, and RA. The general prevalence of MCTD is still unclear [3,4]. For these reasons, the diagnostic classification of MCTD is highly controversial; generally, it is characterized by the presence of U1 antibodies targeting snRNP in the bloodstream. However, there exists significant debate and disagreement regarding how to precisely define and categorize MCTD. Currently, there are four different sets of classification criteria for MCTD, and none of them have received any international consensus or approval. This complexity further complicates the process of diagnosing MCTD. One of challenging issues is differentiating MCTD from other ACTDs, particularly SLE and SSc. In some cases, a patient might initially meet the criteria for MCTD, but upon further evaluation, it becomes evident that they also meet the criteria for another ACTD. As a result, there is a division among rheumatologists: some view MCTD as a distinct and separate disease entity, while others perceive it as a nonspecific stage of development within the spectrum of other ACTDs [5]. In spite of all the ongoing debates over this disease, a comprehensive and extended study involving a substantial patient cohort reveals that the majority of individuals with MCTD exhibit a consistent set of characteristics over the long term. Recent progress in our understanding of MCTD’s underlying mechanisms has proven the pivotal role of anti-U1-RNP autoantibodies. These autoantibodies are mostly found in MCTD patients [3].

Epigenetic alterations, including the modification of DNA and histones, have recently emerged as potential elements in explaining and redefining ACTDs. The best understood DNA modifications are histone acetylation and non-coding RNA (ncRNA) such as micro RNA (miRNA) and DNA methylation. The major role of DNA methylation is the regulation of gene expression. Moreover, this process is a well-characterized epigenetic hallmark for several diseases. DNA methylation is involved in the activity of DNA methyltransferase (DNMT), causing the formation of 5-methylcytosine (5-mC). The methylation of cytosine most commonly affects CpG-rich regions, called CpG islands, which causes the downregulation of expression and results in the silence of a gene function. Abnormal DNA methylation has been observed in autoimmune diseases [6]. What is more, DNA methylation can be one of the possible reasons for ACTDs’ prevalence in females through X chromosome inactivation. It has been shown that females with SLE were characterized by impaired DNA methylation on the inactive X chromosome [7].

Due to MCTD’s low prevalence, there is a significant lack of comprehensive molecular-level investigations into this disease. The fact that there are currently few publications on MCTD reveals a strong need to answer the question of differences and similarities in the pathogenesis of MCTD compared to other ACTDs. Our previous research examined the potential associations between miRNAs related to the immune system, both in their severity and susceptibility to MCTD [8]. Moreover, our research on cell-free microRNA expression profiles that MCTD patients exhibit distinctions from individuals with other autoimmune connective tissue diseases [9]. Some research revealed a widespread hypomethylation pattern affecting genes, with a notable enrichment in functions related to the immune system [10]. Based on the currently available studies on MCTD, it is important to focus on increasing our knowledge of this disease.

Recently, a method often used is the measurement of Global DNA methylation, which refers to the total level of 5-mC content in a sample relative to the total cytosine content. Aberrant gene-specific demethylation and global hypomethylation can potentially lead to the upregulation of gene expression [11]. Global DNA hypomethylation has been described in RA, which may be crucial for the disease pathogenesis [12,13]. Epigenetic states, unlike genetic lesions, are potentially reversible and, hence, candidates for pharmacological intervention [14]. Numerous studies have detailed the manner in which DNA methylation is influenced by the environment, resulting in altered global and gene-specific DNA methylation [15,16,17,18,19]. Indeed, DNA methylation can be influenced by environmental factors such as smoking, diet, drugs, hormones, stress, vitamin D, and periodontitis. It is thought that these environmental factors influence epigenetic modifications, which, in concert with the individual genetic susceptibility status, results in the development of ACTDs’ symptoms [20].

This study was undertaken to define the global DNA methylation changes between MCTD and other ACTDs patients in order to better understand their role in promoting and the course of this disease.

## 2. Results

### 2.1. Global DNA Methylation in ACTDs

**Figure 1:**
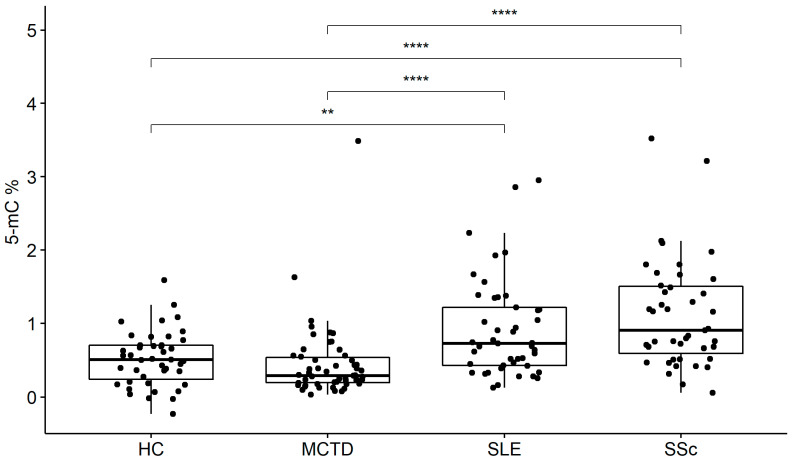
Global methylation 5-mC (%) in systemic lupus erythematosus (SLE), systemic sclerosis (SSc), mixed connective tissue disease (MCTD), and healthy subjects (HC).** *p* < 0.005, **** *p* < 0.00005, ⦁ number of data points.

To search for epigenetic risk factors for ACTDs, we performed a global DNA methylation analysis. The levels of global methylation of DNA in patients with MCTD, SLE, SSc, and HC were demonstrated in Table 1. The highest % of global methylation has been observed in the group of patients with SLE and SSc, whereas with the MCTD patients, healthy subjects were comparable. The present analysis revealed a statistically significant difference of global methylation between SLE and MCTD (*p* < 0.001), SLE and HC (*p* = 0.008), SSc and MCTD (*p ≤* 0.001), and SSc and HC (*p* < 0.001), but neither between MCTD and HC (*p* = 0.09) nor SSc and SLE (*p* = 0.08) (Figure 1).

### 2.2. Global DNA Methylation within SSc Disease

**Figure 2:**
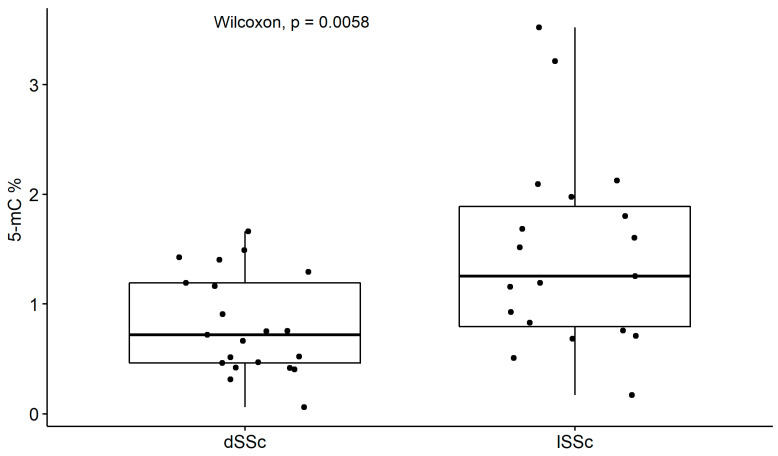
Global methylation 5-mC (%) in clinical subtypes, dSSc—Diffuse SSc; lSSc—limited SSc.

Global DNA methylation was highest among the SSc patients. We assessed whether the methylation levels differed between the clinical subtypes of systemic sclerosis. The analysis showed that patients with limited SSc had significantly higher global methylation levels compared to diffuse SSc patients (*p* = 0.01, Figure 2).

### 2.3. Global DNA Methylation Decrease with Age

**Figure 3:**
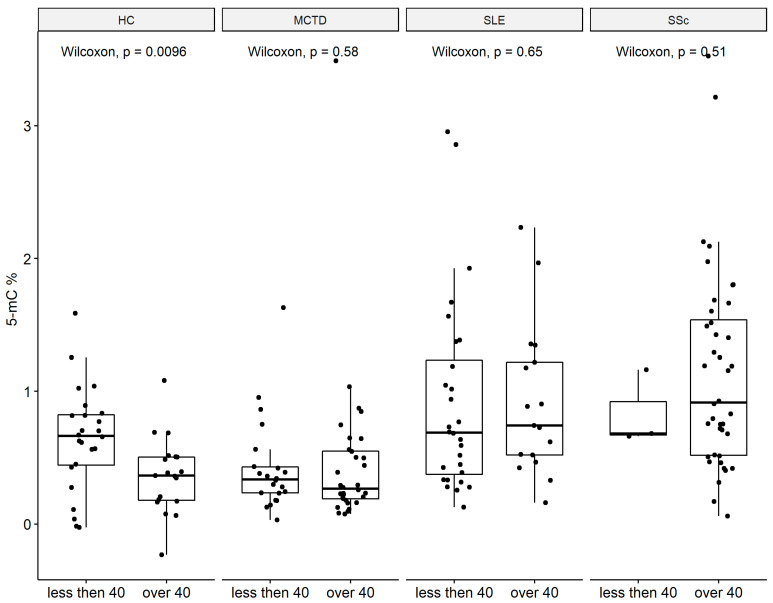
Global methylation 5-mC (%) between age groups. Systemic lupus erythematosus (SLE), systemic sclerosis (SSc), mixed connective tissue disease (MCTD), and healthy subjects (HC). ⦁ number of data points.

We assessed whether the level of global methylation correlated with age. The analysis showed a significant negative correlation only in the control group (r = −0.395, *p* = 0.01). The analysis of methylation levels between patients under 40 years old and over 40 years old showed that, in the control group, older patients had significantly lower levels of global methylation (Mann–Whitney, *p* = 0.01). The level of global methylation did not differ between the age groups in the ACTDs patients (Figure 3).

The present study did not reveal any significant association with clinical parameters in MCTD, SLE, or SSc.

Association analysis of global methylation with main clinical manifestations and disease activity scores was performed. We have not observed any relevant associations. Global methylation was not related to the presentation of any of the studied parameters.

## 3. Discussion

Autoimmune Connective Tissue Diseases are diagnosed based on various laboratory and clinical criteria because their pathogenesis is very complex. These diseases are characterized by clinical heterogeneity with the varied progression of the disease activity, which is a reason for potential failure to establish early diagnosis and appropriate treatment. In general, from the onset of the first symptoms of the disease to an accurate diagnosis can last many years, leading to numerous tissue damages and a bad prognosis. Moreover, some individuals never fulfill the clinical symptoms of a specific systemic autoimmune disease and remain undiagnosed for years or even a lifetime. Despite high heterogeneity, individuals with different ACTDs share some common clinical features. Patients with MCTD may have clinical manifestations observed in SSc, SLE, or RA. Although there are many parameters that identify individual diseases, an overlapping clinical landscape between MCTD and other ACTDs, especially SSc and SLE, still remains very challenging.

Despite the growing knowledge of the importance of epigenetics in the development of autoimmune diseases, there are, to the best of our knowledge, hardly any studies conducted that relate to overlapping syndromes yet, such as MCTD or SLE [8,10,11,12,13,14,15,16,17,18,19,20,21]. Our research is the first study that examined the global DNA methylation level in patients with MCTD, SLE, and SSc. DNA methylation is known to be important for the activity of genes and is specific for cell type, but the exact mechanism in many diseases is still unknown. In general, altered DNA methylation may lead to phenotypic changes. In this study, we evaluated the blood-based DNA methylation levels of SSc, MCTD, and SLE patients. It is worth noting that most of the research on changes in DNA methylation is conducted on PBMCs. We submit that studies requiring blood DNA samples across multiple sites with diagnostic potential should consider DNA from whole blood rather than PBMC, for ease of processing and storage [22]. Moreover, cellular heterogeneity has a potential confounding effect on the outcomes of DNA methylation measurement conducted using whole blood DNA, due to differences in the cellular population. DNA methylation is a tissue-specific process. Glossop et. al. observed changes in methylation within selected genes between B-lymphocytes and T-lymphocytes. Their study provided evidence that the DNA methylation signature is unique to lymphocyte types even in healthy individuals [23,24]. Furthermore, following treatment with MTX, there has been documented evidence of a rise in global DNA methylation levels in T cells, B cells, and monocytes. However, the implications of this phenomenon remain unclear [4,25].

In our study, which aims to distinguish the MCTD group from other ACTD groups, we reported that whole blood DNA from patients with SLE and SSc contained increased amounts of 5-methylocytosine. In contrast to SLE and SSc, patients with MCTD and healthy individuals had decreased DNA methylation levels. The highest level of global methylation has been observed in SSc patients, particularly in the limited the systemic sclerosis subtype. The level of global DNA methylation did not differ between the age groups in ACTDs patients. The patients who participated in this study, excluding patients with SSc, were matched for similar age, so we can exclude the effect of age on the changes in the methylation level. Moreover, the present study did not reveal any significant association with clinical manifestations in ACTDs. We did not detect an association of the global methylation with the main clinical symptoms and the assessment of the disease activity, which may be related to the received corticosteroids and other drugs, which could prevent the detection of some of the clinical parameters of the disease activity. Therefore, the influence of the disease activity on the changes in the DNA methylation level cannot be fully excluded.

Our study provided evidence of global DNA hypomethylation in MCTD patients. Contero-Montoro et al. showed decreased methylated DNA levels in MCTD patients compared to healthy individuals. This difference was observed in genes involved in pathways of type I interferon. Similar to our studies, the authors showed statistically significant differences between MCTDs and other ACTDs, such as RA, SLE, and SSc, which is consistent with our outcomes [10]. Also, Stypinska et al. observed in their study of cell-free microRNA expression profiles that MCTD patients differ from the other ACTDs patients. In the case of their study, there was also no statistically significant difference between patients with MCTD and healthy blood donors [9]. The global methylation levels of DNA were also measured in patients with SLE. Although Liu C. et al.’s research has demonstrated that patients with SLE were characterized by a significantly lower methylation level of DNA than the controls. In our study, the global methylation level of DNA was significantly increased in the SLE patients, in comparison with that in the healthy controls [26]. The discrepancies in the results may be due to ethnic differences. In addition, differences in the global methylation levels may result from the use of another biological material, namely PBMCs [26,27]. The present study has shown that the global DNA methylation level was significantly increased in SSc patients in comparison with the healthy controls. However, other research demonstrated an upward trend in global hypomethylation in SSc [28]. Also, other studies have shown that the global methylation level in SLE and SSc patients was significantly lower than in healthy subjects [29,30]. The etiology of SSc is still unclear. Interestingly, our analysis showed that patients with limited SSc had significantly higher global methylation levels when compared to diffuse patients. DNA methylation profiles may differ between the two subtypes. Ramos et al. compared the DNA methylation levels of 27 pairs of SSc discordant twins, and each subtype of the disease had a different pattern of DNA methylation [31]. Likewise, similar results were obtained by Altorok et al., which showed a different and characteristic DNA methylation pattern displayed in fibroblasts from patients with lSSc and dSSc [32,33].

Due to the clinical diversity of patients and the size of the sample, the present study has some limitations. Differences in the results observed in the available literature may also be associated with group validation or ethnic differences. The limited number of participants in the study, as well as the absence of comprehensive clinical data for certain individuals, may exert an influence on the findings and contribute to the study’s reduced power. Although we are the first to analyze the global DNA methylation level in MCTD patients, the sample size is modest. The overall degree of DNA methylation of a genome can be a useful measure of widespread regulatory changes, but nevertheless reveals a certain view of molecular relationships. MCTD, SLE, and SSc patients involved in this study were mostly under steroids or under antimalarial or immunosuppressive medication at the time of the blood sampling. SLE, SSc, and especially MCTD are rare diseases. The comparison of our results to those previously reported in patients is hindered by differences in the use of a biological material, such as PBMC or fibroblasts [34]. It is worth noting that the degree of DNA methylation is tissue-specific and even varies within B and T lymphocytes. Inherent to all epigenomic studies is the fact that we cannot exclude the possibility of reverse causation, or whether the DNA methylation changes are an effect or a cause of ACTDs. We recognize that it is difficult to account for all factors that could affect the DNA methylation, such as lifestyle, nutrition, medications, body weight, physical activity, environmental stress, etc., which were not accounted for in an adjustment to our study [35]. Nonetheless, further investigation into the DNA methylation level warrants a more extensive and homogenous cohort of patients at a consistent disease stage to draw unequivocal findings. Functional research is imperative to establish a direct connection between the DNA methylation level and the development of MCTD, solidifying the understanding of its underlying mechanisms.

Finally, the present study illustrated for the first time the great potential of the global DNA methylation level of whole blood to discriminate MCTD patients and other ACTDs, which in the future may allow us to unequivocally state the existence or exclusion of MCTD as a separate disease entity and facilitate its diagnosis.

## 4. Materials and Methods

### 4.1. Patients and Clinical Characteristics

The study was conducted on a group of 46 SSc patients, 45 SLE patients, 54 MCTD patients, and 43 healthy controls (HCs). SLE, MCTD, and SSc patients were diagnosed at the Clinic and Polyclinic of Connective Tissue Diseases of the National Institute of Geriatrics, Rheumatology, and Rehabilitation in Warsaw. The control groups consisted of healthy volunteers who do not show any clinical or laboratory signs of autoimmune diseases. Subjects were randomly selected from blood bank donors to match the patients in gender and ethnicity. All participants provided informed written consent for participation in the study. The study was approved by the Ethics Committee of the National Institute of Geriatrics, Rheumatology and Rehabilitation in Warsaw, Poland (14 January 2016).

SLE patients SLE subjects met the American College of Rheumatology/Systemic Lupus international Collaborating Clinics (ACR/SLICC) 2012 classification criteria. Disease activity was examined based on the SLE Disease Activity Index (SLEDAI) score; the damage index was examined based on the Systemic Lupus International Collaborating Clinics/American College of Rheumatology Damage Index (SLICC/ACR DI).

SSc patients SS subjects met ACR/European League Against Rheumatism (EULAR) 2013 classification criteria. Disease activity was examined by the European Scleroderma Research Group (EScSG) AI and DI.

MCTD patients MCTD subjects were eligible in accordance with the classification criteria of Kusakawa and/or Alarcón-Segovia and Villarreal, demonstrating a robust specificity for the diagnosis of MCTD. To evaluate the clinical activity of MCTD, we introduced a novel index called the Mixed Connective Tissue Disease-Activity Index (MCTD-AI). This index was adapted from the activity index used in our institute’s Systemic Lupus Erythematosus Disease Activity Index (SLEDAI). The MCTD-AI incorporates clinical and laboratory symptoms that are indicative of active MCTD. For each symptom observed in the patient over the past 28 days, signifying disease activity, we assigned a specific number of points. The patient’s MCTD-AI score is then calculated as the sum of these points, with a maximum possible score of 52 (for details, see Supporting Information, Table S1). In addition to assessing disease activity, we also developed a MCTD-Damage Index (DI) based on the Systemic Lupus International Collaborating Clinics/American College of Rheumatology (SLICC/ACR) Damage Index. In this index, we assigned one point for each symptom of damage that persisted in MCTD patients for a minimum of 6 months. If an episode of damage recurred, we attributed two points. More information about the specific symptoms and criteria can be found in the Supporting Information, Table S2. These two indicators, the MCTD-AI and the MCTD-DI, were crucial tools in our study to quantitatively assess disease activity and chronic damage in patients with MCTD, providing a valuable framework for understanding and monitoring this complex condition. In our study, we excluded MCTD patients who met the classification criteria for two ACTDs at the time of blood collection.

Patients eligible for the present study were evaluated based on laboratory tests and physical examinations. Disease duration, gender, age, erythrocyte sedimentation ratio (ESR), C-reactive protein (CRP), interstitial lung disease (ILD), forced vital capacity (FCV), high-resolution computed tomography (HRTC), modified-Rodnan skin score (mRSS), autoantibodies profile, and the information about the medication were collected at the time of the clinical material sampling. The age distribution of the patients collected for our study was as follows: MCTD and SLE patients were of similar age, while SSc patients were much older compared to the others. SLE patients had the highest CRP and ESR indicators. The majority of MCTD, SLE, and SSc patients were women, and the percentages were 74.51%, 91.11%, and 67.50%, respectively. The demographic and clinical description of all patients are shown in Table 2.

Healthy subjects included in the present study (20 (47%) women and 23 (53%) men, with a mean age of 39.00 ± 14.76) did not have a history of autoimmune and/or inflammatory disease at the time of sampling.

### 4.2. Global DNA Methylation Assessment

DNA from 500 µL of whole blood was extracted using an AA Biotech Blood Mini (A&A Biotechnology, Gdańsk, Poland) according to the manufacturer’s standard protocol. The quantity and quality of samples were measured with DeNovix (Denovix Inc., Wilmington, DE, USA). The purity of the DNA samples was calculated to a 260/280 nm OD ratio with expected values between 1.8 and 2.0. The DNA samples were stored at −80 °C until required for further analysis. Global DNA methylation levels were analyzed in 80 ng genomic DNA using the ELISA-based commercial kit (MethylFlash Global DNA methylation (5-mC), ELISA Easy Kit (Colorimetric) (EpiGentek Group Inc., Farmingdale, NY, USA) following the manufacturer’s instructions. Briefly, DNA sample was binded to specialized wells with a strong affinity for DNA. The presence of methylated DNA was detected by utilizing specific antibodies that target 5-methylcytosine (5-mC), which is a marker for methylation. Subsequently, we quantified the methylation levels using a colometric approach by measuring the absorbance at 450 nm using a microplate reader Tecan Infinite F PLEX (Tecan Group Ltd., Männedort, Switzerland). The percentage of methylated DNA (5-mC%) in the total DNA sample was calculated using a standard curve generated by the absorbance values of six concentration points (0.1–5%methylated DNA) according to the manufacturer’s instructions. This allowed us to establish a direct proportion between OD intensity and the absolute amount of methylated DNA in the patient sample. A line graph of change in optical density was created using the software MAGELLAN PRO V7.4 STD.2PC.

### 4.3. Statistical Analysis

Normality was checked using the Shapiro–Wilk test and histogram plots. Differences in the global methylation between patient groups were assessed using the Kruskal–Wallis test. Results at a significance level of *p* < 0.05 were considered significant. Post hoc analysis comparing differences between pairs was performed using Bonferroni—Holm adjusted *p* value. Correlation between global methylation and clinical parameters was conducted using Person or Spearman correlation tests. U-Mann–Whitney test or *t*-test was used to analyze differences between two groups. RStudio Version 1.4.1717 © 2023-2021 RStudio, PBC was used to conduct analyses and present graphs. R Packages version 1.3.1 used for data analysis are listed in References section [36,37,38,39,40,41,42].

## 5. Conclusions

In conclusion, our research contributes novel insights into explaining the epigenetic predisposition in individuals with MCTD. Our study provided a basis for further insights into the importance of the global DNA methylation level that differentiated MCTD patients from other ACTDs. Moreover, DNA extracted from whole blood is more readily available for analysis. Further studies into the changes in the epigenetics of ACTD patients may lead to a better understanding of the pathology of these diseases, which in the future can help with establishing the proper diagnosis and appropriate classification of patients, particularly in cases of overlap syndromes. In summary, global DNA hypomethylation may play a multifaceted role in MCTD development, but further research is required to clarify this. It is important to underscore that this study can primarily serve as a direction for subsequent research endeavors or as a reference point for larger-scale investigations into this uncommon medical condition.

## Figures and Tables

**Table 1:** Median (IQR) % of global methylation of DNA in the patients with MCTD, SLE, SSc, and HC.

		Control (N = 43)	MCTD (N = 54)	SLE (N = 45)	SSc (N = 43)
**5-mC (%)**	median (IQR: Q1, Q3)	0.51 (0.24, 0.70)	0.29 (0.20, 0.54)	0.73 (0.43, 1.22)	0.91 (0.59, 1.50)
**age**	mean ± sd	39.00 ± 14.76	43.09 ± 15.27	39.96 ± 13.44	57.28 ± 13.41
**Gender**					
**women**	n (%)	20 (46.51%)	41 (75.93%)	41 (91.11%)	30 (69.77%)
**men**	n (%)	23 (53.49%)	13 (24.07%)	4 (8.89%)	13 (30.23%)

MCTD, mixed connective tissue disease; SLE, systemic lupus erythematosus; SSc, systemic sclerosis; dSSc, diffuse systemic sclerosis; lSSc, limited systemic sclerosis. Continuous variables were presented as median and interquartile range (IQR: Q1, Q3); Categorical variables were presented as percentages.

**Table 2:** Clinical description of patients with MCTD, SSc, and SLE; SSc is divided into two groups: limited (lcSSc) and diffusive (dcSSc).

Parameters	MCTD (N = 51)	SLE (N = 45)	All SSc (N = 43)	dSSc (N = 21)	lSSc (N = 19)
**Age** mean ± sd	44.08 ± 14.92	39.96 ± 13.44	57.00 ± 13.58	57.24 ± 13.41	56.74 ± 14.12
**Gender**					
**women**	38 (74.51%)	41 (91.11%)	27 (67.50%)	15 (71.43%)	12 (63.16%)
**men**	13 (25.49%)	4 (8.89%)	13 (32.50%)	6 (28.57%)	7 (36.84%)
**Disease duration (months)**	116.31 ± 102.75	54.09 ± 84.04			
**Disease activity** median (IRQ)	7 (1.00, 17.00) N = 11	4.00 (2.00, 8.00) * 1.00 (0.00, 2.00) **			
**ILD**			25 (64.10%)	14 (66.67%)	11 (61.11%)
**FVC (%)** mean ± sd			77.48 ± 13.07	75.54 ± 13.41	80.00 ± 12.85
**DLCO** mean ± sd			63.00 ± 14.99	61.44 ± 15.10	64.67 ± 15.21
**HRTC** **0** **2** **5**			14 (38.89%) 12 (33.33%) 10 (27.78%)	7 (35.00%) 6 (30.00%) 7 (35.00%)	7 (43.75%) 6 (37.50%) 3 (18.75%)
**mRSS** median (IRQ)			9.50 (4.00, 13.00)	9.00 (4.75, 12.50)	9.50 (3.50, 13.00)
**CRP** median (IRQ)	5.00 (2.25, 8.23)	8.00 (4.50,19.00)	6.00 (4.00, 9.25)	7.00 (4.00, 10.00)	5.00 (3.50, 8.00)
**ESR** median (IRQ)	15.00 (10.00, 34.75)	19.00 (9.00, 41.50)	17.50 (10.00, 28.25)	16.00 (11.00, 29.00)	18.00 (9.00, 27.50)
**Autoantibody profile**					
**Anti-dsDNA**	2 (5.88%)	29 (65.91%)	1 (3.33%)	1 (5.88%)	0 (0.00%)
**Anti-scl-70**	1 (2.94%)	1 (2.50%)	20 (52.63%)	11 (52.38%)	9 (52.94%)
**Anti Jo-1**	1 (2.94%)	0 (0.00%)	0 (0.00%)	0 (0.00%)	0 (0.00%)
**Anti-histone**	2 (5.88%)	5 (12.82%)	2 (6.06%)	2 (10.53%)	0 (0.00%)
**Anti-Rib-P**	2 (5.88%)	5 (12.82%)	4 (12.50%)	1 (5.26%)	3 (23.08%)
**Anti-Ro/SSA**		17 (43.59%)			
**Anti-Ro/SSA-60**	6 (17.65%)		3 (11.11%)	2 (11.11%)	1 (11.11%)
**Anti-Ro/SSA-52**	8 (23.53%)		7 (18.92%)	5 (25.00%)	2 (11.76%)
**Anti-La/SSB**	3 (8.82%)	6 (15.38%)	0 (0.00%)	0 (0.00%)	0 (0.00%)
**Anti-U1 RNP**	35 (100.00%)	8 (20.51%)			
**Anti-A**	30 (88.24%)				
**Anti-C**	25 (73.53%)				
**Anti-70kD**	24 (70.59%)				
**Anti-nucleosome**			0 (0.00%)	0 (0.00%)	0 (0.00%)
**Anti-Sm**		12 (29.27%)			
**Anti-SmB**	11 (32.35%)		0 (0.00%)	0 (0.00%)	0 (0.00%)
**Anti-SmD**	2 (5.88%)		0 (0.00%)	0 (0.00%)	0 (0.00%)
**Anti-CCP**	4 (8.51%)				
**Anti-PCNA**	1 (2.94%)		0 (0.00%)	0 (0.00%)	0 (0.00%)
**Anti-centromere ACA**			6 (15.79%)	5 (23.81%)	1 (5.88%)
**Anti-CENP-A**			10 (28.57%)	6 (30.00%)	4 (26.67%)
**Anti-CENP-B**		2 (5.00%)	11 (28.21%)	7 (33.33%)	4 (22.22%)
**aCL IgM**		5 (11.90%)			
**aCL IgG**		11 (26.19%)			
**LAC**		14 (35.90%)			
**RF**	25 (51.02%)		5 (14.71%)	4 (20.00%)	1 (7.14%)
**PM_Scl**			4 (11.43%)	3 (14.29%)	1 (7.14%)
**PM-Scl-75**			2 (5.71%)	1 (5.00%)	1 (6.67%)
**PM_Scl_100**			2 (5.71%)	1 (5.00%)	1 (6.67%)
**AMA-M2**			2 (6.45%)	0 (0.00%)	2 (16.67%)
**RP11**			2 (5.88%)	0 (0.00%)	2 (13.33%)
**RP155**			3 (8.33%)	1 (5.00%)	2 (12.50%)
**Anti-Fibrillarin**			4 (11.43%)	1 (5.00%)	3 (20.00%)
**Anti-NOR 90**			1 (2.86%)	0 (0.00%)	1 (6.67%)
**Anti-Th/To**			1 (2.86%)	0 (0.00%)	1 (6.67%)
**Anti-Ku**			2 (5.56%)	2 (10.00%)	0 (0.00%)
**Anti-PDGFR**			0 (0.00%)	0 (0.00%)	0 (0.00%)
**Medication**	Methotrexate −14%	Methotrexate −17%		Methotrexate −23%	Methotrexate −26%
		Steroids −97%		Steroids −14%	Steroids −15%
	Immunosuppressive drugs −24%	Azathioprine −37%		Immunosuppresive drugs −95%	Immunosuppresive drugs −73%
	Chloroquine −16%	Chloroquine −45%		Vasodilators −95%	Vasodilators −89%
	Hydroxychlorquine −5%	Hydroxychlorquine −37%		Amlodipine—85%	Amlodipine −89%
	Cyclophoshamid −9%	Cyclophoshamid −10%			

MCTD, mixed connective tissue disease; SLE, systemic lupus erythematosus; SSc, systemic sclerosis; dSSc, diffuse systemic sclerosis; lSSc, limited systemic sclerosis; ILD, interstitial lung disease; FVC, forced vital capacity; DLCO, diffusing capacity of the lung of carbon monoxide; HRTC, high-resolution computed tomography, 0-normal, 2-ground-glass opacification, 5 changes made (reticular or fibrosis); mRSS, modified-Rodnan skin score; RF, rheumatoid factor; anti-CCP, anti-cyclic citrullinated peptide autoantibodies; anti-Scl-70, anti-topoisomerase 1; anti-dsDNA, anti-double stranded DNA; CRP, C-reactive protein; ESR, erythrocyte sedimentation rate; SSA, Sjogren’s-syndrome-related antigen. Continuous variables were presented as median and interquartile range (IQR); Categorical variables were presented as percentages * SELENASLEDAI; ** SLICC.

## Data Availability

The authors confirm that the data supporting the findings of this study are available from the corresponding authors (A.P-G) on request.

## Supplementary Materials

The following supporting information can be downloaded at: https://www.mdpi.com/article/10.3390/ijms242015495/s1.

## Abbreviations

5-mC	5-methylocytosine
ACA	Anti-centromere antibody
aCL IgG	Anti Cardiolipin antibody of immunoglobulin G
aCL IgM	Anti Cardiolipin antibody of immunoglobulin M
ACR	American College of Rheumatology
ACTDs	Autoimmune Connective Tissue Diseases
AMA-M2	Anti-mitochondrial M2 antibody-positive autoimmune hepatitis
ANGPT2	Angiopoietin 2
Anti-CCP	anti-cyclic citrullinated peptide autoantibodies
Anti-CENP-A	Anti-centromere proteins A
Anti-CENP-B	Anti-centromere proteins B
Anti-dsDNA	Anti-double stranded DNA
Anti Jo-1	Anti-nuclear antibody
Anti-La/SSB	Anti-SLE Sjogren’s syndrome or SLE-related autoantibodies
Anti-PCNA	Proliferating cell nuclear antigen antibody
Anti-Rib-P	Anti-ribosomal P
Anti-Ro/SSA	Anti-Sjogren’s-syndrome-related antigen A autoantibodies
Anti-Scl-70	Anti-topoisomerase I
Anti-Sm	Anti-Smith
Anti-SmB	Anti-Smith B
Anti-SmD	Anti-Smith D
Anti-U1 RNP	Anti-U1RNP antibody
CRP	C-reactive protein
DLCO	Diffusing capacity of the lung of carbon monoxide
DNMT	DNA methyltransferase
DNMT3a	DNA methyltransferase 3 Alpha
DNMT3b	DNA methyltransferase 3 Beta
dSSc	diffuse systemic sclerosis
ESR	Erythrocyte sedimentation rate
EULAR	European Alliance of Associations for Rheumatology
FCV	Forced vital capacity
HDAC4	Histone deacetylase 4
HRTC	High-resolution computed tomography
ILD	Interstitial lung disease
LAC	Lupus anticoagulant antibody
lSSc	lLmited systemic sclerosis
MCTD	Mixed connective tissue disease
miRNA	Micro RNA
mRSS	Modified-Rodnan skin score
MTX	Methotrexate
MVEC	Microvascular endothelial cells
ncRNA	Non-coding RNA
NOS1	Nictric oxide synthase 1
PBMC	Peripheral blood mononuclear cell
RF	Rheumatoid factor
SLE	Systemic lupus erythematosus
SSc	Systemic sclerosis

## References

[1] Karagianni P., Tzioufas A.G. (2019). Epigenetic perspectives on systemic autoimmune disease. J. Autoimmuny.

[2] Wu H., Chang C., Lu Q. (2020). The Epigenetics of Lupus Erythematosus. Adv. Exp. Med. Biol..

[3] Venables P.J.W. (2006). Mixed connective tissue disease. Lupus.

[4] Tani C., Carli L., Vagnani S., Talarico R., Baldini C., Mosca M., Bombardieri S. (2014). The diagnosis and classification of mixed connective tissue disease. J. Autoimmuny.

[5] Kasukawa R. (1999). Mixed connective tissue disease. Intern. Med..

[6] Hurtado C., Acavedo Saenz L.Y., Vasquez Trespalacios E.M., Urrego R., Jenks S., Sanz I., Vasquez G. (2020). DNA methylation changes on immune cells in Systemic Lupus Erythematosus. Autoimmunity.

[7] Steward J.J. (1998). The female X-inactivation mosaic in systemic lupus erythematosus. Immunol. Today.

[8] Stypinska B., Lewandowska A., Felis-Giemza A., Olesińska M., Paradowska-Gorycka A. (2021). Association study between immune-related miRNAs and mixed connective tissue disease. Arhtritis Res. Ther..

[9] Stypinska B., Wajda A., Walczuk B., Olesinska M., Lewandowska A., Walczyk M., Paradowska-Gorycka A. (2020). The serum Cell-Free microRNA expression profile in MCTD, SLE, SSc, and RA patients. J. Clin. Med..

[10] Carnero-Montoro E., Barturen G., Povedano E., Kerick M., Martinez-Bueno M., Ballestar E., Martin J., Teruel M., Alarcon-Riquelme M. (2019). Epigenome-Wide Comparative Study Reveals Key Differences between Mixed Connective Tissue Disease and Related Systemic Autoimmune Disease. Front. Immunol..

[11] Fouad M.A., Salem S.E., Hussein M.M., Zekri A.R., Hafez H.F., Desouky E.D., Shourman S.A. (2018). Impact of Global DNA Methylation in Treatment Outcome of Colorectal Cancer Patients. Front. Pharmacol..

[12] Nakano K., Whitaker J.W., Boyle D.L., Wang W., Firestein G.S. (2013). DNA methylome signature in rheumatoid arthritis. Ann. Rheum. Dis..

[13] Karouzakis E., Gay R.E., Michael B.A., Gay S., Neidhart M. (2009). DNA hypomethylation in rheumatoid arthritis synovial fibroblasts. Arthritis Rheum..

[14] Szyf M. (2010). Epigenetic therapeutics in autoimmune disease. Clin. Rev. Allergy Immunol..

[15] Martin E.M., Fry R.C. (2018). Environmental Influences on the Epigenome: Exposure-Associated DNA Methylation in Human Populations. Annu. Rev. Public. Health.

[16] Agodi A., Barchitta M., Quattrocchi A., Maugeri A., Canto C., Marchese A.E., Vinciguerra M. (2015). Low fruit consumption and folate edficiency are associated with LINE-1 hypomethylation in women of cancer-free population. Genes. Nutr..

[17] Breton C.V., Byun H.M., Wenten M., Pan F., Yang A., Gilliland F.D. (2014). Prenatal tabacco smoke exposure affects global and gene-specific DNA methylation. Am. J. Respir. Crit. Care Med..

[18] Carmona J.J., Sofer T., Hutchinson J., Cantone L., Coull B., Maity A., Vokonas P., Lin X., Schwartz J., Baccarelli A.A. (2014). Short-term airborne particulate matter exposure alters the epigenetic landscape of human genes associated with the mitogen-activated protein kinase network: A cross-sectional study. Environ. Health.

[19] Beach S.R.H., Lei M.K., Ong M.L., Brody G.H., Dogan M.V., Philibert R.A. (2017). MTHFR methylation moderates the impact of smoking on DNA methylation at AHRR for African American young adults. Am. J. Med. Genet. B Neuropsychiatr. Genet..

[20] Cribbs A., Feldmann M., Oppermann U. (2015). Towards an understanding of the role of DNA methylation in rheumatoid arthritits therepeutic and diagnostic implications. Ther. Adv. Musculoskelet. Dis..

[21] Kreuz-Imgenberg J., Almlof J.C., Leonard D., Alexsson A., Nordmak G., Eloranta M.L., Rantapaa-Dahlqvist S., Bengtsson A.A., Jonsen A., Padyukov L. (2018). DNA methylation mapping identifies gene regulatory effects in patients with systemic lupus erythematosus. Ann. Rheum. Dis..

[22] He D., Yang C.X., Sahin B., Shannon C.P., Oliveria J.P., Gavreau G.M., Tebbutt S.J. (2019). Whole blood vs PBMC: Compartmental differences in gene expression profiling exemplified in asthma. Allergy Asthma Clin. Immunol..

[23] Adalsteinsson B.T., Gudnason H., Aspelund T., Harris T.B., Launer L.J., Eiriksdottir G., Smith A.V., Gudnason V. (2012). Heterogeneity in white blood cells has potential to confound DNA methylation measurements. PLoS ONE.

[24] Glossop J.R., Nixon N.B., Emer R.D., Haworth K.E., Packham J.C., Dawes P.T., Fryer A.A., Mattey D.L., Farrell W.E. (2013). Epigenome-wide profiling identifies significant differences in DNA methylation between matched-pairs of T- and B-lymphocytes from healthy individuals. Epigenetics.

[25] De Andres M.C., Perez-Pampin E., Calaza M., Santaclara F.J., Ortea I., Gomez-Reino J.J., Gonzalez A. (2015). Assessment of global DNA methylation in peripheral blood cell subpopulations of early rheumatoid arthritis before and after methotrexate. Arthritis Res. Ther..

[26] Liu C.C., Ou T.T., Wu C.C., Li R.N., Lin Y.C., Lin C.H., Tsai W.C., Liu H.W., Yen J.H. (2011). Global DNA methylation, DNMT1, and MBD2 in patients with systemic lupus erythematosus. Lupus.

[27] Wu H., Zhao M., Tan L., Lu Q. (2016). The key culprit in the pathogenesis of systemic lupus erythematosus: Aberrant DNA methylation. Autoimmun. Rev..

[28] Dal-Bekar N.E., Siomek-Gorecka A., Gackowski D., Koken-Avsar A., Yarkan-Tugsal H., Birlik M., Islekel H. (2022). Global hypomethylation pattern in systemic sclerosis: An application for absolute quantification of epigenetic DNA modification products by 2D-UPLC-MS/MS. Clin. Immunol..

[29] Matatiele P., Tikly M., Tarr G., Gulumian M. (2015). DNA methylation similarities in genes of black South Africans with systemic lupus erythematosus and systemic sclerosis. J. Biomed. Sci..

[30] Lei W., Luo U., Lei W., Luo Y., Yan K., Zhao S., Li Y., Qiu X., Zhou Y., Long H. (2009). Abnormal DNA methylation in CD4+ T cells from patients with systemic lupus erythematosus, systemic sclerosis, and dermatomyositis. Scand. J. Rheumatol..

[31] Ramos P.S., Zimmerman K.D., Haddad S., Langefeld C.D., Medsger T.A., Feghali-Bostwick C.A. (2019). Integrative analysis of DNA methylation in discordant twins unveils distinct architectures of systemic sclerosis subsets. Clin. Epigenetics.

[32] Frost A., Silveira W., Hazard E., Atanelishvili I., Wilson R., Flume J., Day K., Oates J., Bogathevich G., Feghali-Bostwick C. (2021). Differential DNA methylation landscape in skin fibroblasts from African Americans with systemic sclerosis. Genes.

[33] Altorok N., Tsou P., Coit P., Khanna D., Sawalha A.H. (2015). Gemone-wide DNA methylation analysis in dermal fibroblasts from patients with diffuse and limited systemic sclerosis reveals common and subset-specific DNA methylation aberrancies. Ann. Rheum. Dis..

[34] Nyce J. (1989). Drug-induced DNA hypermethylation and drug resistance in human tumors. Cancer Res..

[35] Lim U., Song M.A. (2012). Dietary and lifestyle factors of DNA methylation. Methods Mol. Biol..

[36] Wickham H., Bryan J. (2019). Readxl: Read Excel Files. R Package Version 1.3.1. https://CRAN.R-project.org/package=readxl.

[37] Wickham H., Francois R., Henry L., Muller K. (2021). dplyr: A Grammar of Data Manipulation. R Package Version 1.0.7. http://CRAN.R-project.org/package=dplyr.

[38] DeWitt P. (2021). qwraps2: Quick Wraps 2. R Package Version 0.5.2. https://CRAN.R-project.org/package=qwraps2.

[39] Wickham H. (2016). ggplot2: Elegant Graphics for Data Analysis.

[40] Kassambara A. (2020). ggpubr: ‘ggplot2’ Based Publication Ready Plots. R Package Version 0.4.0. https://CRAN.R-project.org/package=ggpubr.

[41] Fox J., Weisberg S. An {R} Companion to Applied Regression.

[42] Dinno A. (2017). dunn.test: Dunn’s Test of Multiple Comparisons Using Rank Sums. R Package Version 1.3.5. https://CRAN.R-project.org/package=dunn.test.

