# Supplementary material for: Mixed Connective Tissue Disease as Different Entity: Global Methylation Aspect
Source: Int J Mol Sci. 2023 Oct 23;24(20):15495. doi: 10.3390/ijms242015495 (PMC10607312; doi:10.3390/ijms242015495)
Supplement: Supplementary file 1 [file ijms-24-15495-s001.zip › ijms-2591989-supplementary.pdf]

Supplementary Material

Table 1S. MCTD activity (MCTD-AI) scale.

Point value	Clinical manifestation
8	Pulmonary hypertension
8	Pulmonary involvement
8	Vasculitis
4	Myositis
4	Esophageal involvement
4	Neuropathy
4	Kidney involvement
2	Puffy hands
2	Serositis
2	Skin symptoms
2	Polyarthritis
1	↑ ESR/CRP
1	↑gamma globulins
1	↓ Hb
1	Platelets<br>↓leucocytes

Table 2S. MCTD damage (MCTD-DI) scale.

Type of symptoms	Clinical manifestation
Kidney involvement	Proteinuria > 3,5g/24h
	End-stage renal disease
	GFR < 50 %
Cardiovascular symptoms	Cardiomyopathy / ventricular dysfunction
	Hypertension (HA) treated > 6 months
	Coronary artery disease or coronary artery bypass
	Pericarditis > 6 months / pericardiectomy
	Valve dysfunction
	Myocardial infarction
Eye symptoms	Cataract
	Changes in the retina or optic atrophy
Neuropsychiatric symptoms	Stroke
	Neuropathy of the cranial or peripheral nerves (excluding the optic nerve)
	Cognitive impairment
	Transverse myelitis
	Seizures requiring treatment > 6 months
Lung involvement	↓ DLCO
	↓ FEV1
	"Shrinking lung" syndrome
	Pulmonary fibrosis (confirmed by X-ray / CT / biopsy)
	Pulmonary hypertension (> 10 % from the upper limit of normal)
Gastrointestinal symptom	Chronic peritonitis
	Infarction / intestine or other abdominal organ / resection of the stomach
	Impaired gastrointestinal motility, diarrhea, abdominal pain, or constipation
	Dysfunction – esophageal dysfunction documented by X-ray
Skin symptoms	Skin ulcers (excluding thrombosis) for at least 6 months
	Skin calcification / subcutaneous tissue / soft tissue
	Extensive scarring or skin atrophy (except for scalp and fingertips)
	Fat loss (local / diffuse)
	Alopecia with scarring
	Deforming or erosive arthritis
	Osteoporosis with peripheral / compression fractures of the vertebrae (excluding aseptic

Musculoskeletal symptoms	necrosis)
	Muscle atrophy (reduction of muscle mass assessed by clinical examination)
	Muscle weakness not resulting from active muscle disease
	Non-fracture osteoporosis requiring anti-osteoporotic treatment (except calcium and vitamin D3 supplementation)
	Aseptic necrosis
Endocrine Disorder	Hypertrichosis
	Irregular periods
	Diabetes
	Growth retardation (children)
	Delayed development of secondary sex characteristics (children)
	Sexual dysfunction (adults)
	Infertility (adults)
	Primary or secondary amenorrhea
Infections	Multiple infections (>3 infections at the same / different location within 6 months)
	Chronic infections
Tumor	Cancer (specify type, stage)
Others	Death (cause, date)
